# Clinical and functional analyses of the novel STAR c.558C>A in a patient with classic lipoid congenital adrenal hyperplasia

**DOI:** 10.3389/fgene.2023.1096454

**Published:** 2023-01-17

**Authors:** Jie Liu, Hong-Mei Dai, Gao-Peng Guang, Wen-Mu Hu, Ping Jin

**Affiliations:** ^1^ Department of Endocrinology, The Third Xiangya Hospital of Central South University, Changsha, Hunan, China; ^2^ Department of Pediatric, The Third Xiangya Hospital, Central South University, Changsha, China

**Keywords:** star steroidogenic acute regulatory protein, lipoid congenital adrenal hyperplasia, congenital lipid adrenal hyperplasia, mutation, classic LCAH

## Abstract

**Objective:** Congenital lipid adrenal hyperplasia (LCAH) is the most serious type of congenital adrenal hyperplasia and is caused by steroid-based acute regulatory (STAR) protein mutations. Herein, we report compound heterozygous mutations c.558C>A (p.S186 R) and c.772C>T (p.Q258*) in a newborn 46 XY patient diagnosed with classic LCAH and explore their clinical and functional characteristics.

**Methods:** Peripheral blood samples were collected from LCAH patient and their families. The pathogenic variant identified by whole-exome sequencing was further confirmed by Sanger sequencing and pedigree verification. The functional consequence and ability to convert cholesterol into progesterone of the identified *STAR* Q258* and S186 R mutations were analyzed by cell transfection and *in vitro* assays.

**Results:** The proband was presented with severe glucocorticoid and mineralocorticoid deficiency, high adrenocorticotropic hormone, and enlarged adrenals. Heterozygous mutations p. S186 R and p. Q258* in the *STAR* gene were identified in the patient, and her parents were carriers, which is consistent with an autosomal recessive disorder. The *STAR* p. Q258* mutation has been reported and generates a truncated protein. The p. S186 R mutation is a novel variant that disrupts STAR. The residual STAR activities of p. S186R, p. Q258*, and p. S186R/p.Q258* were 13.9%, 7.3%, and 11.2%, respectively, of the wild-type, proving the main negative effects of the mutant proteins.

**Conclusion:** Our findings reveal the molecular mechanisms underlying LCAH pathogenesis, further expanding the genotype and clinical spectrum of LCAH.

## Introduction

Congenital adrenal hyperplasia (CAH) is a group of autosomal recessive disorders mainly caused by pathogenic variations in genes encoding steroidogenesis enzymes and cofactors. The most common CAH is a 21-hydroxylase deficiency caused by *CYP21A2* gene mutation (El-Maouche et al., 2017). Other pathogenic variations involved in the biosynthesis of glucocorticoids can also lead to rarer CAH, such as *STAR, CYP11A1, HSD3B2, CYPB11 B1, CYP17A1,* an*d POR* (Merke and Auchus, 2020).

Steroidogenic acute regulatory (STAR) protein (OMIM #600617) can limit the inflow of cholesterol from the extramitochondrial membrane to the internal membrane of mitochondria (Miller and Bose, 2011; Manna et al., 2016), where lateral chain cutting enzyme (P450scc) can convert cholesterol into progeste. Thus, loss of StAR function affects the first step of steroidogenesis, inhibiting all steroid production in adrenal glands and gonads, leading to the most serious form of CAH, lipoid congenital adrenal hyperplasia (LCAH, OMIM 201710) (Kaur et al., 2016; Ullah et al., 2020). Most LCAH result from loss-of-function mutations in the double allele *STAR* gene, while a few are caused by *CYP11A1* gene mutations (Sahakitrungruang et al., 2011; Pomahačová et al., 2016). LCAH can be divided into classic and non-classic forms (Garg et al., 2020), with the classic form characterized by severe glucocorticoid and mineral corticosteroid deficiency at birth, as well as female or minimally male external genitalia regardless of the sex chromosome. In contrast, non-classic LCAH patients depict milder phenotypes, with late adrenal cortical insufficiency and male external genitalia in XY males (Ishii et al., 2020; Luo et al., 2020).

Herein, we report two heterozygous mutations *STAR* c.558C>A (p.S186R) and c.772C>T (p.Q258*) in a 46 XY patient diagnosed with classic LCAH and explore its clinical and genetic characteristics.

## Materials and methods

### Patient data and ethics statement

This study was approved by the Institutional Ethics Committee of Third Xiangya Hospital and conformed to the principles outlined in the Declaration of Helsinki. Written informed consent was obtained from all participants to collect peripheral blood samples of LCAH patients and their families. DNA was extracted from peripheral blood for Sanger sequencing.

### Whole-exome sequencing

All exons were sequenced in the proband to identify causal genes. The DNA was cut into a length of 200–300 bp on the Bioruptor UCD-200 (Diagenode), diluted, loaded, and sequenced on the HiSeq2500 platform (Illumina, San Diego, CA). Exome data processing and variant annotation were performed as previously described (Hu et al., 2020; Zhang et al., 2020; Guan et al., 2022), focusing on LCAH pathogenic genes. Mutations are rare events with a frequency of less than 1% in 1,000 genomes (http://browser.1000genomes.org), Exome Aggregation Consortium (ExAC, http://exac.broadinstitute.org/), and Genome Aggregation Database (gnomAD, http://gnomad.broadinstitute.org/). The variants were interpreted according to the American Society of Medical Genetics (ACMG) standards and classified as pathogenic, potentially pathogenic, or meaningless variants that may be benign.

### Sanger sequencing

Sanger sequencing was performed to identify potential pathogenic variations. The primers of the STAR gene exon five were: forward GGA​GAG​CCC​AGT​GTG​AAT​GC; reverse ATC​TTG​TCT​TTG​TCC​CTC​CTT​TGG. The ABI 3730 xl automated sequencer (Applied Biosystems, USA) was used to identify mutations.

### In silico analysis

The effects of single nucleotide variants (SNVs) were predicted using SIFT (http://sift.jcvi.org), PolyPhen-2 (http://genetics.bwh.harva rd. edu/pph2), MutationTaster (http://www.mutationtaster.org), and PROVEAN (http://provean.jcvi.org/index.php) programs. The STAR amino acids of different species were aligned using AlignX software (Invitrogen). The mutated residues were mapped to the crystal structure of the STAR complex to evaluate the structure and/or functional effects of mutations, and PyMOL (https://pymol.org/) was used to prepare molecular graphic images.

### Plasmids

Wild-type (WT) full-length STAR cDNA (NM_000349.3) containing HindIII and BamHI restriction endonuclease sites was synthesized into plasmid carrier pcDNA 3.1 (+). The p. Q258*, p. S186R, and p. Q258*/p.S186R mutations were present in the STAR cDNA inserted into the pcDNA3.1 plasmid using the *Mut Express* ^®^ II Fast Mutagenesis kit V2 System (Vazyme, China). Plasmid generation was confirmed by Sanger sequencing by Hefei Bionei Biotechnology Co., Ltd (China). The pcDNA3.1 plasmid was purchased from Shanghai Jikai Gene Chemical Technology Co., Ltd (China). The cDNA sequences of P450scc, adrenodoxin, and adrenodoxin reductase were obtained from NCBI (https://www.ncbi.nlm.nih.gov/nuccore/). The cDNAs of P450scc, adrenodoxin, and adrenodoxin reductase were chemically synthesized *in vitro*, and the cDNA sequences were separately cloned into the pc3.1 (+) plasmid, which is called F2.

### Cell culture and transfection

Monkey kidney fibroblast-derived COS-7 cells were purchased from Pnoise Life Technology Co., Ltd (China) and cultured in Dulbecco’s modified Eagle/F12 medium (DMEM/F12, HyClone, United States) supplemented with 10% fetal bovine serum (FBS; Life Technologies) at 37°C and 5% CO_2_. When the cells reached 80% confluence, they were seeded in a six-well plate and grown to 60%–70% confluence. The cells were then transfected with the plasmids using Lipofectamine3000 (Invitrogen, United States) at a transfection ratio of 1:2, and the culture medium was changed after 24 h.

### RT-PCR analysis

The 1 μg each of pcDNA3.1, WT, S186R, Q258*, Q258*/S186R-STAR plasmids, and lip3000 were transfected into Cos7 cells at a ratio of 1:2. After 48 h of transfection, the total RNA of the transfected Cos7 cells was extracted using TRIzol (Takara, Japan), quantified using a NanoDrop spectrophotometer (United States), and reverse-transcribed using the First Strand cDNA Synthesis kit (Toyobo, Japan) and oligo-dT primers on a Roche LightCycler 480 II Real-Time PCR System (Roche, United States). Relative gene expression was determined by the comparative method (2^−ΔΔCT^) using a KOD SYBR qPCR Mix Kit (Toyobo, Japan) and gene-specific primers: Star forward, AGA​CTT​CGG​GAA​CAT​GCC​TGA​G-3; reverse, GAC​CTG​GTT​GAT​GAT​GCT​CTT​GG; GAPDH forward, GCC​TTC​CGT​GTT​CCT​ACC; reverse, GCC​TGC​TTC​ACC​ACC​TTC.

### Western blotting

The 1 μg each of pcDNA3.1, pcDNA3.1-WT, pcDNA3.1-S186R, pcDNA3.1-Q258*, pcDNA3.1-Q258*/S186 R -STAR plasmids and Lip3000 were transfected into Cos7 cells at a ratio of 1:2. Total protein was extracted from transfected Cos7 cells and quantified using the BCA assay (Nanjing Kechuang Biotechnology Co., Ltd., China). Proteins were separated by SDS-PAGE and transferred to nitrocellulose membranes using a transfer device (Bio-Rad, Hercules, CA, United States). The membranes were blocked with 5% skimmed milk for 2 h and then incubated with the primary antibodies, STAR and GAPDH (Proteintech, Chicago, IL, United States) diluted to 1:1,000 in TBS buffer overnight at 4°C. After washing with TBS, the membranes were incubated with goat IgG rabbit polyclonal antibodies (1:2,000 in TBS, Proteintech) for 2 h. After washing three times with TBS buffer, the protein bands were visualized using ECL Western blotting luminal reagent (Advansta, Menlo Park, CA, United States) on a universal Hood II chemiluminescence detection system (Bio-Rad).

### Enzymatic activity assay

The 1 μg each of pcDNA3.1 empty plasmid, WT-STAR, S186R, Q258*, and Q258*/S186R-STAR plasmids, together with 1ug F2 plasmid, were transfected into Cos7 cells. A 24 h after replacing the transfection medium, it was replaced with a culture containing 22 (R)-hydroxycholesterol (5 μg/ml). After 48 h, the supernatant was collected to detect STAR-dependent pregnenolone production using a progesterone ELISA kit (AlPCO, United States).

## Results

### Clinical characteristics of CAH patient

A 2.5-month-old girl was referred to our hospital for recurrent vomiting, diarrhea, and dehydration. She was the second child of healthy consanguineous parents, with a birth weight of 4.2 kg. Physical examination revealed hyperpigmented skin around the hands, lips, and nipples. She had a normal external female sex. Biochemical tests revealed hyponatremia (117 mmol/L), hyperkalemia (5.5 mmol/L), and metabolic acidosis (Table 1). Her serum adrenal corticosteroid (ACTH) > 2000 pg/mL with low levels of cortisol (0.3 ng/ml) and aldosterone (23.6 pg/mL). All adrenal steroid levels, including those of testosterone, dehydroepiandrosterone sulfate, androstenedione, and 17-hydroxyprogesterone, were extremely low (Table 1). Chromosomal genetic testing demonstrated a 46 XY male karyotype. Ultrasound examination demonstrated testicular tumors in the pelvic cavity (1.4 cm * 0.5 cm * 0.5 cm on the left and 1.0 cm * 0.6 cm * 0.5 cm on the right), and there were no visible ovaries or uterus. Adrenal computed tomography (CT) revealed enlargement and reduced density of the bilateral adrenal glands. Her parents and an eight old sister were healthy. She was initially diagnosed with LCAH and prescribed hydrocortisone (3.75 mg) and 9 a-fludrocortisone (0.1 mg) orally. During follow-up, the steroid replacement dose was adjusted according to growth and laboratory examination results (Table 1). The child grew and developed normally, pigmentation was slightly reduced, and there was no subsequent adrenal crisis. She is currently treated with hydrocortisone 6.75 mg/day and has stopped fludrocortisone. ACTH and blood electrolyte levels were within the normal range (Table 1).

**TABLE 1:** Clinical data of the patient with LCAH before and after treatment.

	2 m	4 m	7 m	10 m	1 y 2 m	1 y 6 m	2 y	2 y 4 m	RR
Ht (cm)	57.6	61.8	67.2	70.7	75.2	79.2	85.0	88.4	
Wt (cm)	5.2	6.2	9.7	10.5	9.9	11.8	12.0	13.7	
Na^+^ (mmmol/L)	117	131.5	141.2	139.8	146.2	141.7	138.7	140.7	137.0–147.0
K^+^ (mmol/L)	5.5	6	3.9	4.2	4.3	4.1	4.5	3.9	3.5–5.3
Cortisol (ng/mL)	0.3	0.5	0.4	0.3	0.7	0.6	0.6	0.8	4.8–19.3
ACTH (pg/mL)	>2000	>2000	997.9	49.8	118.7	44.1	14.9	14.9	6–48
Aldosterone (pg/mL)	23.6	29.7	10.5	—	—	17.4	13.7	13.4	40–310
Renin activity (ng/mL/h)	9.3	4.2	2.44	—	—	3.7	7.4	—	1.31–3.95
DHEAS (µg/mL)	0.6	0.6	0.2	—	—	0.02	—	—	0.26–5.85
17-OHP (ng/mL)	0.2	0.01	0.1	—	—	0.03	—	—	≤1.47
AD (pg/mL)	0.02	0.02	0.02	—	—	0.02	—	—	0.06–0.78
Testosterone (ng/mL)	0.03	0.04	0.09	—	—	0.02	—	—	≤2.01
Hc (mg)	3.75	6.25	6.25	5.8	6.25	5.8	7.5	6.75	
9 *a*-Fc (mg)	0.1	0.15	0.15	0.15	0.05	—	—	—	

Ht, height, Wt, weight; DHEAS, dehydroepiandrosterone; 17α-OHP, 17OH-progesterone; AD, androstenedione, Hc, hydrocortisone; Fc, fludrocortisone; RR, reference range.

### Mutation analysis of the *STAR* gene

**FIGURE 1:**
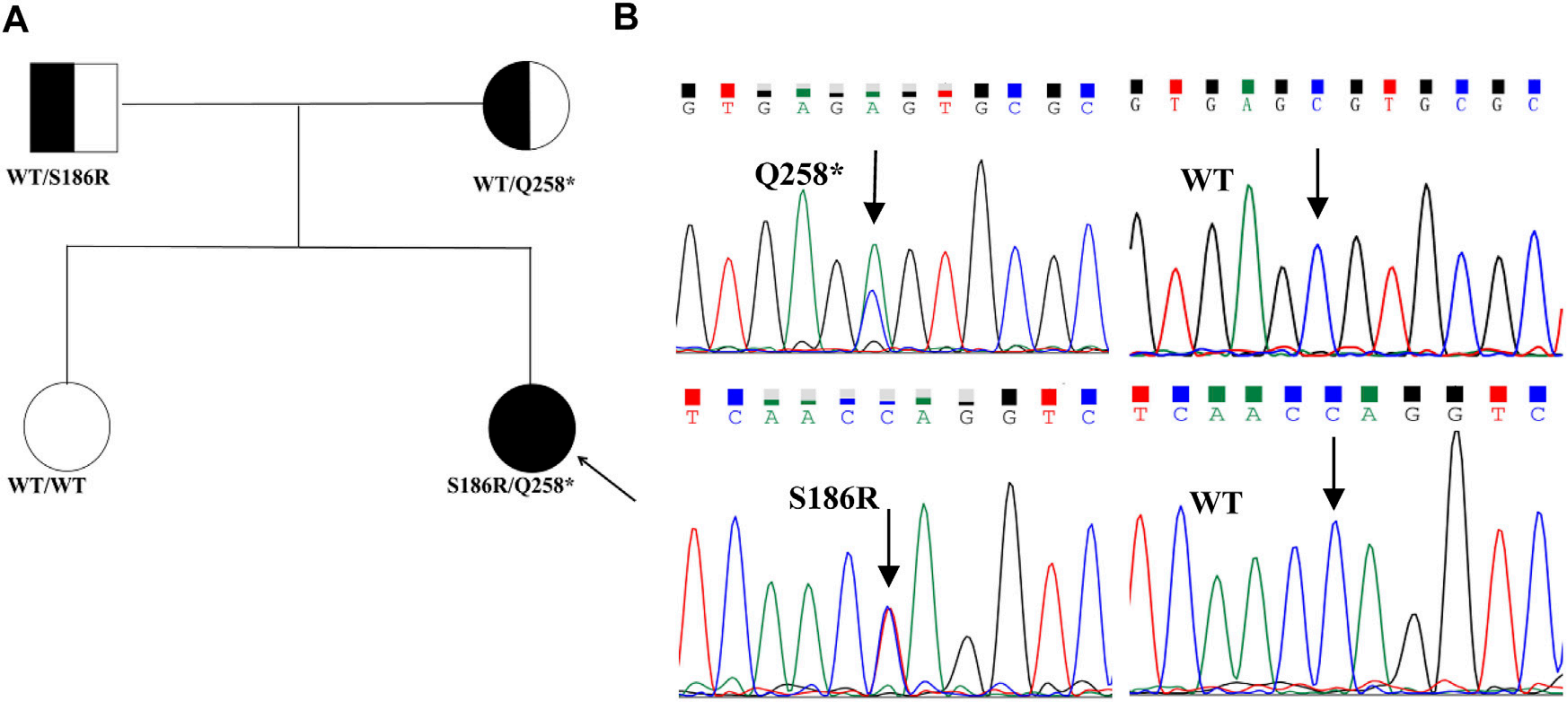
Pedigrees and sequencing chromatograms of the mutations identified in the families. **(A)** Family pedigrees, squares, and circles indicate males and females, respectively. Shaded symbols indicate the proband (indicated by the arrow). The half-shaded symbols indicate carriers **(B)**. The heterozygous STAR c558C>A (p.S186R) and c.772C>T (p.Q258*) mutations were identified in the proband. The arrow indicates the mutation site.

The proband had heterogeneous variations in *STAR* c.558C>A (p.S186R) and c.772C>T (p.Q258*), as verified by Sanger sequencing (Figure 1B). Co-segregation analysis demonstrated that the c.558C>A (p.S186R) variant was paternally inherited and the c.772C>T (p.Q258*) variant was maternally inherited (Figure 1A), both of which had a normal phenotype consisting of an autosomal recessive mode of inheritance. The *STAR* c.772C>T (p.Q258*) non-sense mutation has been previously reported and is predicted to produce an early termination codon in exon 5, resulting in truncated proteins. The STAR c.558C>A (p.S186R) missense mutation causes the 186th amino acid to change from Ser to Arg, which does not exist in control (1,000 Genomes, ExAC, gnomAD, and CNGB) and is designated as “probably damaging” by the PolyPhen-2 software, “damaging” by the SIFT software, and “disease-causing” by the Mutation Taster software. The Human Gene Mutation Database (HGMD) and literature analysis suggest that c.558C>A (p.S186R) is a novel variant.

### Structural analyses of the mutations

**FIGURE 2:**
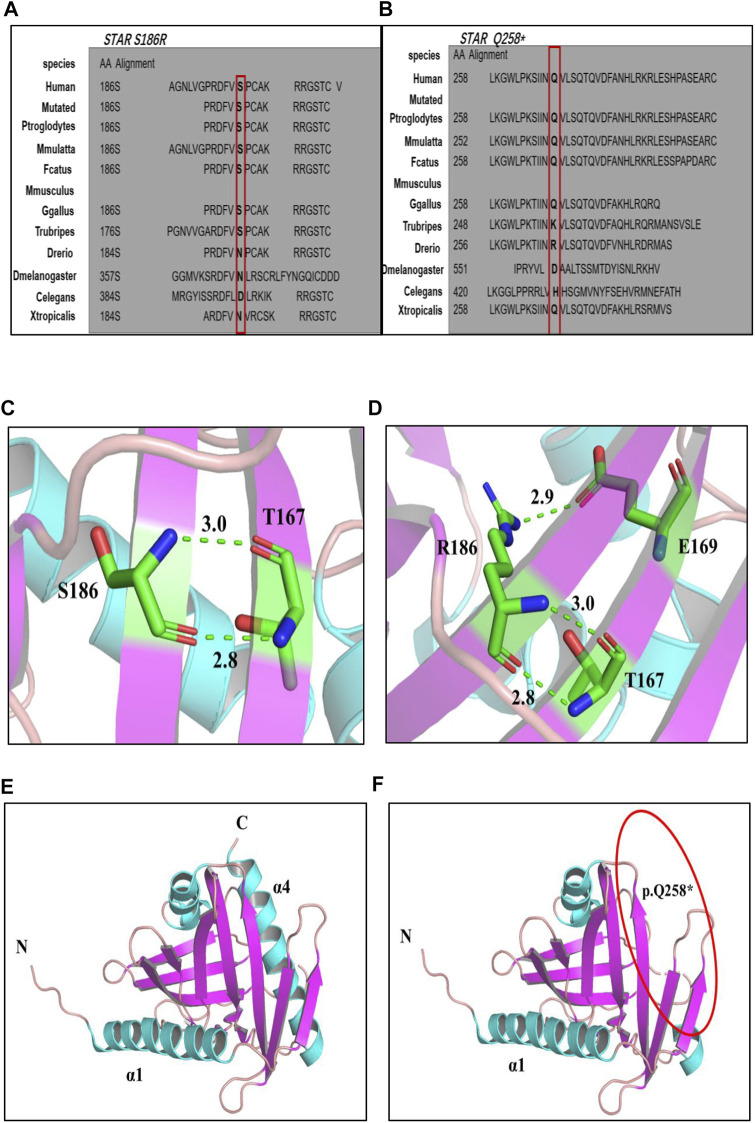
**(A, B)**: Multiple alignment of the STAR protein sequence in different species, indicating conservation of the residues STAR p. S186R **(A)** and p. Q258* **(B)** affected by these mutations. **(C–F)** 3D structures of wild-type and mutant STAR proteins. **(C, D)** Wild-type and p. S186 R STAR protein; **(E, F)** Wild-type and p. Q258*STAR protein. Notes: helix: sky blue; *β*-sheet: purple; α-loop: pink; C atom: green; H atom: gray; N atom: ocean blue; O atom: red; H2O: orange; hydrogen bond: green dotted line.

Amino acid sequence comparison of the STAR gene between different species revealed that the 258th glutamine and 186th serine are conserved (Figures 2A, B). The p. S186R and p. Q258* mutations were mapped onto the three-dimensional (3D) structure of STAR proteins (Figures 2C–F), which was determined using X-ray crystallography. 3D model analyses revealed that after Ser→Arg on amino acid 186 is replaced; a new hydrogen bond is formed between amino acid E (Glu) 169 and R (Arg)186, which changes the local conformation of the cholesterol-binding pocket. Q258* generates a truncated protein at position 258, which disrupts the C-terminal α4 structure required for lipids to enter the binding pocket. Based on protein conservation and 3D structure analyses, it can be concluded that these two mutations may affect the stability and activity of the protein.

### Functional analysis of the STAR mutation *in vitro*

**FIGURE 3:**
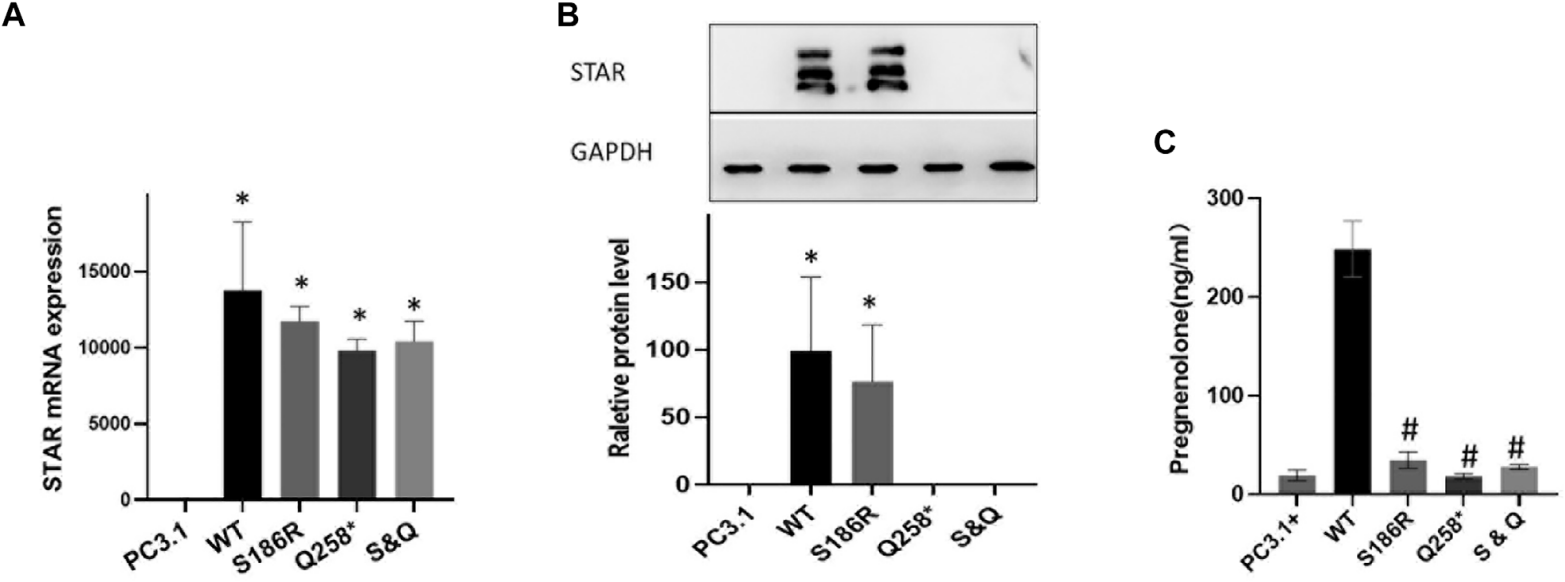
Functional analysis of the STAR mutation *in vitro*. **(A)** STAR mRNA expression of the mutant Q258*, S186R, and Q258*/S186R was similar to wild-type STAR but higher than the empty plasmid vector. **(B)** The protein expression of the mutant S186R was similar to that of the wild-type STAR but greater than the empty plasmid vector. The mutant p. Q258* and Gln258X/S186R were too low to detect. **(C)** Progesterone expression: mutation Q258*, S186R, and Q258*/S186R compared with the wild-type significantly reduced after cholesterol stimulation *p < 0.05 vs empty pcDNA3.1 plasmid; #*p* < 0.05 vs pcDNA3.1-WT-STAR plasmid.

Plasmids containing wild-type or mutated Q258*, S186R, or Q258*/S186R-STAR were transfected into COS-7 cells. First, STAR mRNA and protein expression levels were compared using RT-PCR and western blotting, respectively. The results demonstrated that there was no significant difference in mRNA levels between the wild-type and STAR mutants, but was significantly higher than that of the empty plasmid vector (Figure 3A). The results of western blotting demonstrated that compared to the wild-type, the p. S186R mutation in STAR was normal, whereas the p. Q258* and Q258*/S186 R mutations were too low to detect (Figure 3B). Furthermore, COS-7 cells were transfected with plasmids expressing wild-type or mutant STAR to study the functional consequences of these identified mutations and evaluate their ability to convert cholesterol into progesterone. The results demonstrated that STAR-dependent pregnenolone production by cells transfected with Q258*, S186 R, and Q258*/S186R was significantly reduced compared to that in the wild-type (*p* < 0.05, Figure 3C). The Q258*, S186R, and Q258*/S186R-STAR mutations were 7.3%, 13.9%, and 11.2% of the wild-type protein activity, respectively. The relative activity of the empty vector was 6.5% of that of the wild type, indicating STAR-independent steroidogenesis.

These results indicate that *STAR* Q258* and S186R severely affect STAR-dependent pregnenolone production, which proves the dominant-negative effect of the mutant proteins. According to ACMG guidelines, the *STAR* p. Q258* mutation is pathogenic, given that this mutation was a previously established pathogenic variant (PS1), a null variant (PVS1) with extremely low frequency (PM2), and was co-segregated. The novel S186R mutation is also interpreted as pathogenic because, according to functional analysis, the mutation has a destructive effect on transcription activation (PS3), is predicted to be damaging and disease-causing by multiple computational programs (PP3), has an extremely low frequency (PM2) and is co-segregated in multiple affected family members (PP1).

## Discussion

LCAH is the rarest and most severe type of CAH and is caused by homozygous or compound heterozygous mutations in the *STAR* gene. In this study, we describe the case of a newborn 46 XY patient diagnosed with classic LCAH. This patient presented with severe glucocorticoid and mineralocorticoid deficiency, high adrenocorticotropic hormone levels, and enlarged adrenals. Although our patient had an XY karyotype, he presented with complete female external genitalia, with testes hidden inside the pelvic cavity. As previously reported, the fertility of LCAH is different in severely affected 46 XY and 46 XX babies. The 46 XY patients depict female external genitalia because of the severe inhibition of masculinization during the fetal period, whereas the 46 XX patients may present with spontaneous puberty, breast development, and menstruation due to STAR-independent estrogen synthesis (Tanae et al., 2000; Miller, 2017). However, anovulatory menstruation and impaired fertility may occur because of insufficient progesterone synthesis and lipid droplet infiltration in the ovary (Ishii et al., 2016). The mechanism of classic LCAH is explained by a two-hit model (Bose et al., 1996). The first hit was the loss of STAR-mediated steroidogenesis, which resulted in the accumulation of cholesterol esters in steroidogenic cells. The second hit was that the accumulated lipid droplets eventually destroyed all the remaining steroidogenic capacity. Early diagnosis and replacement of steroid hormones are effective in reducing mortality. LCAH Children have no accumulation of metabolic intermediates and do not need to inhibit hydrogenate, the therapeutic dose of hydrocortisone may be lower than that of children with 21-hydroxylase deficiency to avoid growth inhibition.

The *STAR* gene consists of seven exons and encodes a 285 amino acid protein with a mitochondrial leader sequence at its N-terminus and a highly conserved STAR domain (from 67 to 280) on its COOH-terminus, which are key sites for binding cholesterol and other lipids (Clark, 2012). Crystallography modeling demonstrated that the STAR domain forms an α/β helix-grip fold with a nine-stranded anti-parallel *ß*-sheet, forming a U-shaped hydrophobic pocket that binds lipids (Iyer et al., 2001; Romanowski et al., 2002; Thorsell et al., 2011). Arakane et al. (Arakane et al., 1996) found that deleting up to 62 residues from the N-terminus did not significantly affect steroidogenesis, whereas the removal of 28 C-terminal amino acids inactivated STAR and the deletion of C-terminal 10 amino acids reduced steroidogenic activity by half, which indicated that residues in the C-terminus are essential for steroidogenesis-enhancing activity. To date, more than 80 mutations have been identified in *STAR* (HGMD). Missense mutations are the most common, followed by frameshift, splicing, and non-sense mutations. Most mutations are clustered in exons 4–7, with exon five having the largest number of mutations (Ishii et al., 2020).

Our patient harbored compound heterozygous mutations p. S186R and p. Q258* in the *STAR* gene, and her parents had carrier status, which is consistent with an autosomal recessive disorder. Q258* generates a truncated protein that disrupts the C-terminal α4 structure, which is required for the entry of lipids into the binding pocket. Q258* has been documented as a founder mutation in the Japanese and Korean populations (Bhangoo et al., 2005; Kang et al., 2017) and is also prevalent in Chinese patients with LCAH (Zhang et al., 2021). The novel p. S186R mutation is located within the key sites of the START domains and is predicted to be pathogenic *in silico*. Structural analyses suggested that the Ser→Arg substitutions on residue 186 destroyed the local conformation of the cholesterol-binding pocket. Interestingly, Zhang et al. (2021) described another mutation (p.S186G) at the same site and found that pregnenolone synthesis in p. S186G mutants had almost no residual activity. Some studies (Baker et al., 2006; Sahakitrungruang et al., 2010; Flück et al., 2011) have also described other mutations (p.V187M, p. R188C, and p. R192C) located in the same region and found that the p. V187M, p. R188C, and p. R192C mutant STAR proteins retained only 28%, 17%, and 39.4% of STAR activity, respectively, confirming that the S186R mutation is located in a biologically significant area. As previously reported, if the activity of the mutant protein is less than 10% of the wild-type, the clinical symptoms are more typical of LCAH (Hasegawa et al., 2000). Functional *in vitro* studies found that the residual SAR activities of p. S186R, p. Q258*, and p. S186R/p.Q258* were 13.9%, 7.3%, and 11.2% of those of the wild type, respectively, which is consistent with the phenotype of classic LCAH in our patient.

In conclusion, we identified novel compound heterozygous *STAR* mutations, p. S186R and p. Q258*, in classic LCAH. These findings shed light on the molecular mechanisms underlying the pathogenesis of LCAH and broaden the known genotypic and clinical spectrums of LCAH.

## Data Availability

The raw data supporting the conclusion of this article will be made available by the authors, without undue reservation.
